# Cell-extrinsic controls over neocortical neuron fate and diversity

**DOI:** 10.1126/sciadv.adw0218

**Published:** 2025-09-17

**Authors:** Natalia Baumann, Ilaria Morassut, Sergi Roig-Puiggros, Esther Klingler, Giorgia Bartolini, Sabine Fièvre, Denis Jabaudon

**Affiliations:** ^1^ Department of Basic Neurosciences, University of Geneva, Geneva, Switzerland.; ^2^ Department of Genetics and Evolution, University of Geneva, Geneva, Switzerland.; ^3^ Clinic of Neurology, Geneva University Hospital, Geneva, Switzerland.; ^4^ Université Paris Cité, Imagine Institute, Paris, France.

## Abstract

Neocortical cellular diversity emerges gradually during development. Cell-extrinsic interactions shape this extended maturation, yet cell type–specific dependence on such cues has not been systematically examined. To address this, we compared how cell identity and diversity unfolds in different conditions during neocortical development. Conditions were modified in vivo using genetically modified mouse models in which position or innervation is altered and in vitro using two-dimensional cultures. This approach revealed a molecular hierarchy in which cell class–distinguishing features emerge first, followed by subclass- and type-related characteristics. Acquisition of cellular identity and diversity remained stable across in vivo models. In contrast, in vitro glutamatergic neurons showed decreased expression of identity-defining genes, reduced diversity, and alterations in connectivity. Cellular identity and diversity were closest to in vivo values in organotypic cultures. These findings reveal population-specific responses to environmental conditions and highlight the role of extracellular context in shaping cell diversity in the maturing neocortex.

## Teaser

Cell-extrinsic cues are key for neocortical cell identity and diversity.

## INTRODUCTION

The neocortex consists of multiple cell populations whose identity can be defined by unique combinations of morphologies, spatial arrangements, connectivity, and gene expression (*1*). Understanding how neuronal identity emerges during development is important because transcriptional programs ultimately relate to specific connectivity and circuit function.

The neocortex has a particularly prolonged developmental period compared to other brain structures: In many species, it is still immature at birth, as evidenced by ongoing cell migration and myelinization and incomplete circuit formation (*2*). Cell-extrinsic factors, including input-dependent factors, are thought to influence cell fate during critical periods of postnatal development (*3*), as some cortical neuron types appear particularly sensitive to external stimuli. For instance, layer 2/3 (L2/3) neurons in the primary visual cortex need visual input to acquire their identity (*4*), L4 neurons in the primary somatosensory cortex require tactile stimulation to organize into whisker-related barrels (*5*), and some subtypes of GABAergic neurons depend on activity for migration (*6*). However, for most cell populations, the extent to which cell-intrinsic factors contribute to cell fate and diversity remains unknown. Such data are critical as many neuropsychiatric disorders, including autism spectrum disorder, are thought to emerge through complex interactions between genetic and cell-extrinsic factors (*7*–*9*).

A comprehensive evaluation of cell-extrinsic controls over cell type–specific fate is thus essential. While previous studies have identified differences between in vivo and in vitro conditions (*10*, *11*), here, we address this question by providing a systematic transcriptomic analysis of these differences during postmitotic neocortical maturation using a hierarchical cell identity framework.

A major limitation in quantifying cell-extrinsic effects across cell populations is the difficulty in defining and normalizing responses across cell types. To address this limitation, we first determined the transcriptional identities of mature cortical cells, which we then used to build a hierarchical reference framework to classify query cells following experimental manipulation of cellular context. We used multiple paradigms to evaluate the impact of these manipulations on identity and diversity: in vivo studies in Reeler mouse mutants with abnormal cellular positioning (*12*), VB^−^ mice with disrupted thalamocortical innervation (*5*), and in vitro investigations using two-dimensional (2D) cultures and organotypic slice cultures.

We systematically examined how changes in cellular context affected cell fate. While cellular identity and diversity remained stable in in vivo models, this stability was not maintained in vitro. Identity-defining transcriptional programs were particularly sensitive to in vitro conditions, leading to a loss of cellular diversity, with glutamatergic neurons being most affected.

In organotypic slice cultures, cellular identity and diversity reached levels comparable to those observed in vivo. Together, our findings uncover neocortical cell population–specific sensitivities to extrinsic cues and highlight the critical role of extracellular context in establishing cellular diversity during postnatal development.

## RESULTS

### Cell population–specific maturation dynamics in the neocortex

**Fig. 1.**
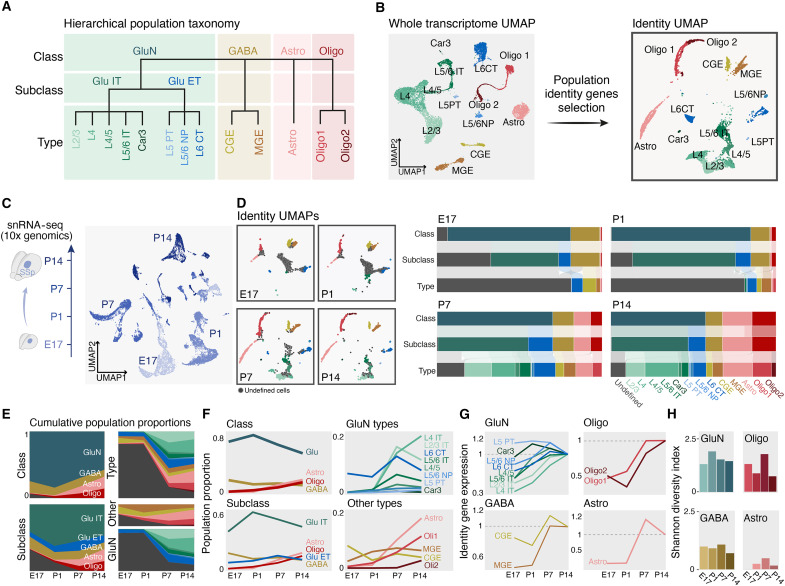
An identity-based framework to annotate cell identity. (**A**) Hierarchical tree containing 4 cell classes, 5 cell subclasses, and 13 cell types built by iterative clustering of the Allen Brain single-cell RNA sequencing dataset [see fig. S1 (A and B)] (*13*). (**B**) UMAP representation of the cell types of the reference dataset (left) and its associated identity UMAP calculated on population identity genes (right). (**C**) Schematic of experimental design (left) and UMAP representing the sequenced nuclei at the indicated time points (right). (**D**) Identity UMAPs at each indicated time point. Cell types color-coded as in (A) and (B). Undefined cells, labeled in gray, decrease as development progresses. The Sankey diagrams on the right summarize the relative proportions for each hierarchical level per time point and early emergence of class definition. (**E**) Cumulative proportions (*y* axis) for classes and subclasses across the four time points analyzed (*x* axis) (left). On the right, the same type of plot is presented for types (top) and for glutamatergic neurons (bottom) separated from the other types (middle). (**F**) Population proportions for classes and subclasses across the four time points analyzed (*x* axis) (left). On the right, the same type of plot is presented for glutamatergic types (top) and for other types (bottom) separately. (**G**) Average relative expression of identity genes for corresponding cell classes across the four time points analyzed. (**H**) The four bar plots display the values of the Shannon index for each cell class separately across the four time points analyzed. GluN, glutamatergic neurons; GABA, GABAergic neurons; Astro, astrocytes; Oligo, oligodendrocytes; Glu IT, glutamatergic intratelencephalic neurons; L6 CT, layer 6 corticothalamic neurons; PT, pyramidal tract neurons; NP, near-projecting neurons; CGE, caudal ganglionic eminence; MGE, medial ganglionic eminence; ID, identity.

Neocortical cells can be hierarchically categorized into classes, subclasses, and types (together, cell populations) based on their molecular identities (*1*). Classes are the broadest category, essentially distinguishing neuronal and non-neuronal cells, while types correspond to the finest-grained classification. To benchmark the developmental emergence of this organization, we first determined the mature hierarchy of cells in the adult mouse primary somatosensory cortex (SSp). Using unbiased iterative clustering of neocortical cells at incremental resolutions, we analyzed a comprehensive single-cell transcriptomic dataset (Fig. 1A, fig. S1, A and B, and Materials and Methods) (*13*). We identified four cell classes, corresponding to glutamatergic neurons, GABAergic neurons, astrocytes, and oligodendrocytes. At the next hierarchical level, five subclasses emerged, which, within glutamatergic neurons, distinguished intratelencephalic (IT) and extratelencephalic (ET) neurons. Last, the highest-resolution level corresponded to a repertoire of 13 cell types, including eight types of glutamatergic neurons. At this clustering resolution, GABAergic neurons comprised two types, as did oligodendrocytes, while astrocytes belonged to a single population across hierarchical levels. The Oligo1 type expressed marker genes of oligodendrocytes precursor cells and maturing oligodendrocytes (e.g., *Pdgfr*α), while the Oligo2 type expressed markers of myelinating oligodendrocytes (e.g., *Mbp*) (table S1). The greater diversity of glutamatergic neurons observed here reflects our specific analytical framework and sampling parameters, not an inherent biological property, and other cell parameters such as electrophysiology or morphology are not taken into account in this classification. This iterative clustering technique showed similar grouping of cells when performed on our own postnatal day 14 (P14) in vivo dataset, indicating a similar hierarchy of population at this stage of maturation (fig. S1B).

A cell’s transcriptome dynamically reflects its state, influenced by both intrinsic and extrinsic factors. While some transcriptomic components are shared across populations, others are expressed specifically, constituting “identity”-defining transcriptional programs. To delineate these identity programs and distinguish features unique to specific cell populations, we identified differentially expressed genes at each hierarchical level (Materials and Methods). This allowed us to unbiasedly identify population identity genes, which categorize cells into classes, subclasses, and types (*n* = 1566 class-specific transcripts, *n* = 1534 subclass-specific transcripts, and *n* = 3452 type-specific transcripts; table S1). Using these identity genes, we generated a corresponding “identity UMAP” (Uniform Manifold Approximation and Projection) in which cells distribute according to their average expression of the identity genes (Fig. 1B and fig. S1C). Within this reference space, query cells can be assigned a class, subclass, and type identity based on the identity of their nearest referenced neighbors or remain undefined when they do not reach a predefined threshold of proximity to single populations (Fig. 1D, fig. S1D, and Materials and Methods).

We assessed the developmental emergence of class/subclass/type hierarchy in this reference space. To this end, we microdissected the putative SSp on embryonic day 17.5 (E17.5), P1, P7, and P14 and performed single-nucleus RNA sequencing (snRNA-seq) (Fig. 1C). This strategy uncovered a hierarchical acquisition of mature cellular identities: Classes were defined first, followed by subclasses and types. This maturation extended into the second postnatal week and was cell type specific, with glutamatergic neurons reaching mature identities later than the other types (Fig. 1E). This difference may reflect the greater diversity of glutamatergic neurons compared to other classes within our classification framework, as well as asynchronous starting points for analysis—GABAergic neurons are more mature than glutamatergic neurons when we start our analysis since they were born at a remote location. Glutamatergic neurons displayed type-specific developmental dynamics, with a sharp increase in IT-defined neurons between P1 and P7, which was reflected in the expression dynamics of their identity-defining genes (Fig. 1, F and G). Cellular diversity, as measured using the Shannon index, which quantifies the evenness and richness of categories in a population, was notable in neurons relative to other classes (Fig. 1H) (*14*). These data reveal a hierarchical molecular program governing cortical cell differentiation, with class-distinguishing features emerging first, followed sequentially by subclass- and type-related characteristics, consistent with progressive specification of neuron fate (*11*).

### Cell type–specific sensitivity to extracellular context in Reeler and VB^−^ mice

**Fig. 2.**
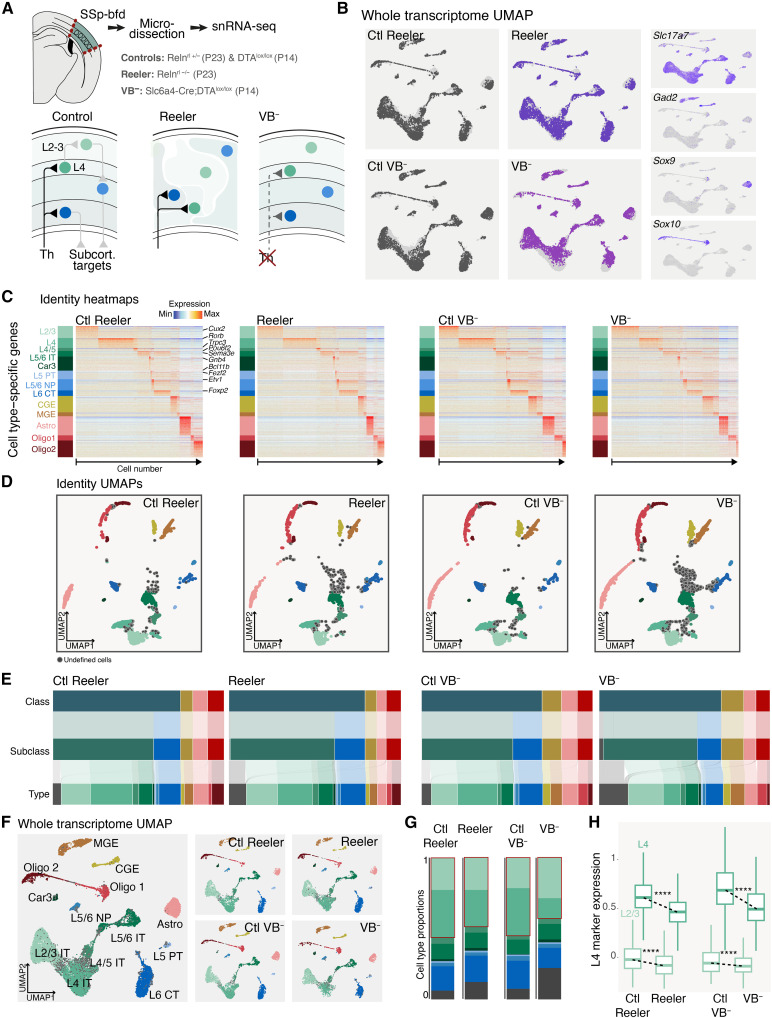
In vivo perturbation of laminar position or thalamocortical input affects specific aspects of cellular identity. (**A**) Schematic representation of snRNA-seq dataset generation for the P23 Reeler and P14 VB^−^ mouse model and their respective controls. (**B**) UMAP representation of snRNA-seq datasets color-coded per condition using whole transcriptome data. On the right, feature plots for four class-specific markers are displayed. (**C**) Heatmaps representing the cell type identity gene expression in Reeler and VB^−^ mice and their respective controls. (**D**) Identity UMAPs representing cell type classification for the Reeler model and its respective control (left) and for VB^−^ and its respective control (right). Undefined cell types are color-coded in dark gray. (**E**) Sankey diagrams showing the relative proportions of the cellular populations identified at each of the three hierarchical levels, represented separately for each of the analyzed conditions. (**F**) Whole transcriptome UMAP for all conditions together (left) and split per condition (right). (**G**) Proportions of glutamatergic neuron types across the four analyzed conditions. The red rectangles highlight the proportion of L2/3 and L4 IT types. (**H**) Boxplots showing expression levels of L4 marker genes within L2/3 IT neurons (light green) and L4 IT neurons (dark green) across the four conditions. ****P < 0.0001. SSp-bfd, primary somatosensory cortex, barrel fields; Th, thalamus; Ctl, control.

To examine cell-extrinsic influences on cell fate, we used transgenic mouse models in which extracellular context is perturbed. To address the role of cellular position, we used Reeler mice, in which the laminar disposition of cells within the cortex is altered (*12*); to evaluate the role of synaptic input, we used VB^−^ mice, in which primary sensory thalamic input to SSp is lacking (Fig. 2A) (*5*). We then analyzed the cell populations at each hierarchical level in these models and their respective controls using snRNA-seq (Fig. 2, B to D, and fig. S2, A and B) at mature stages, when neuronal identity and diversity are fully established (see Fig. 1, D and E). In both Reeler and VB^−^ mice, all 4 classes, 5 subclasses, and 13 types were present (Fig. 2E and fig. S2, C and D), with strong preservation of cellular diversity in both models (fig. S2E). Both mutants displayed more cells with undefined identities, predominantly positioned between L2/3 IT and L4 IT neuron clusters (Fig. 2F). This was more pronounced in VB^−^ mice, consistent with these cell types being the main target of the ventrobasalis (VB) nucleus of the thalamus (*5*, *15*). VB^−^ mice displayed a shift in the proportion of these neuron types, with an increase in L2/3 IT neurons at the expense of L4 IT neurons (Fig. 2G). This finding, coupled with the reduced definition of L4 IT identity in both mutants (Fig. 2H), aligns with previous research suggesting an input-dependent emergence of L4 IT neurons from L2/3 IT-like neurons in SSp (*16*). This suggests strong cell-autonomous controls over cell type–specific transcriptional properties, highlighting the robustness of intrinsic transcriptional programs in maintaining cell identity and diversity. Of note, previous studies have shown that functional circuit connectivity remains largely preserved in both models, with maintained columnar organization and topological maps in Reeler mice (*12*, *17*–*19*) and compensatory rewiring in VB^−^ mice (*5*), supporting a corresponding robustness in cortical circuit formation.

### Cell population–specific molecular responses in 2D cultures

**Fig. 3.**
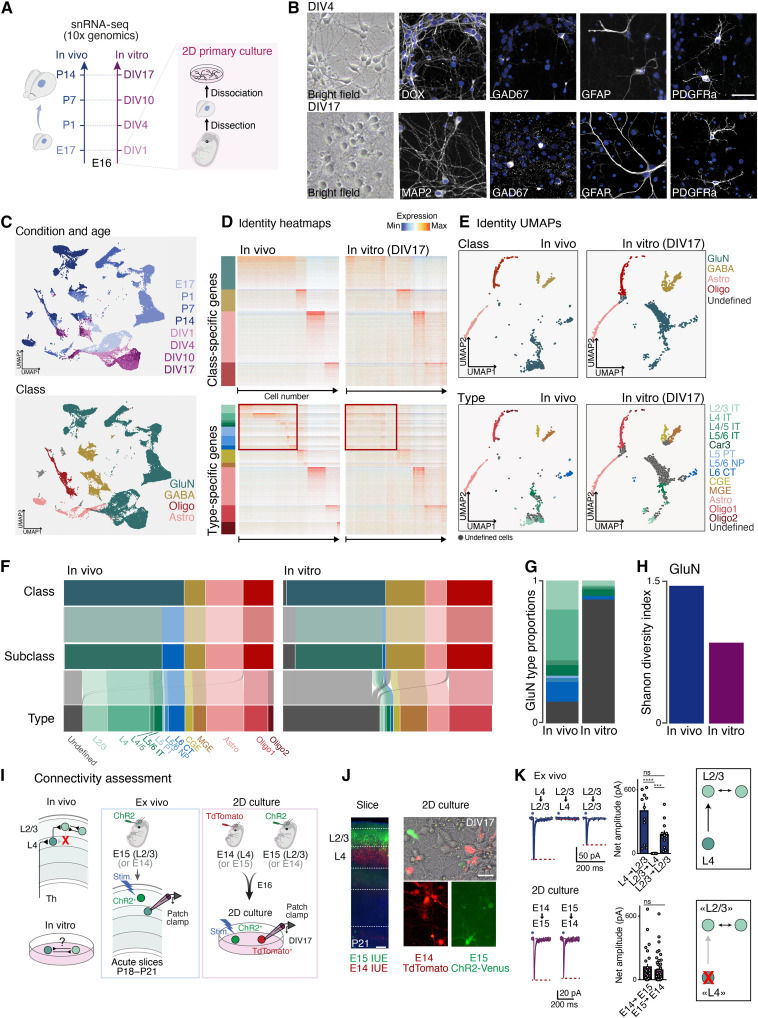
Glutamatergic neuron identity is the most sensitive to environmental conditions. (**A**) Experimental design. (**B**) Left: Representative images of DIV4 and DIV17 cultures**.** Right: Immunofluorescence of DIV4 and DIV17 cultures with glutamatergic (DCX, MAP2), GABAergic (GAD67), astrocytic [glial fibrillary acidic protein (GFAP)], and oligodendrocyte precursor (PDGFRa) markers. Scale bar: 50 μm. (**C**) UMAP representation of snRNA-seq dataset color-coded by time point (top) and cell class (bottom). (**D**) Heatmaps displaying expression of identity makers for cell class and type genes (rows) for each cell (columns), in vivo (left) and in vitro (right). The red boxes indicate gluN-type gene expression within gluN-type cells. (**E**) Identity UMAPs color-coded per class (top) and types (bottom), for in vivo (left) and in vitro (right). (**F**) Sankey diagrams showing the relative proportions of cell populations at each hierarchical level, between P14 (left) and DIV17 (right). (**G**) Relative proportions of glutamatergic types, including undefined (gray), between P14 and DIV17*.* (**H**) Shannon index for in vivo and in vitro glutamatergic neurons. (**I**) Experimental scheme for connectivity assessment. (**J**) Left: Example image of P21 cortex, electroporated at E14 with CAG-TdTomato and at E15.5 with CAG-GFP plasmid. Scale bar, 100 μm. Right: Example image of mixed cultures used for connectivity experiment. (**K**) Left: Example of current traces. Middle: Barplot of net amplitudes measured for each condition. Right: Schematic of the circuit connectivity measured in vivo at P18 to P21. GluN, glutamatergic neurons; Stim., stimulation; ChR2, channelrhodopsin 2; IUE, in utero electroporation; ns, not significant. ***P < 0.001, and ****P < 0.0001.

In vivo, compensatory mechanisms may mask potential extrinsic effects on cellular identity. To uncover such effects, we turned to an in vitro model. We assessed emergence of cellular identities with snRNA-seq in 2D cultures of E16 dissociated SSp at days in vitro (DIVs) 1, 4, 10, and 17, comparing to corresponding ages in vivo, i.e., E17 (corresponding to E16 + 1 day), P1 (E16 + 4 days), P7 (E16 + 10 days), and P14 (E16 + 17 days) (Fig. 3, A to C). In this environment, all cell classes were able to emerge (Fig. 3B), with neurons exhibiting normal resting membrane potential and action potential firing, suggesting adequate culture conditions (fig S3A).

In vitro class identities were similar to those observed in vivo (Fig. 3, C and D). Relative proportions of classes were, however, altered, with a decrease in glutamatergic neuron fraction (Fig. 3, E and F), suggesting that this class is more dependent on extrinsic cues for their survival or differentiation. All five subclasses were well delineated (Fig. 3F and fig. S3, B and C), but the proportion of ET glutamatergic neurons was strongly reduced. To assess ET neuron survival, we labeled ET and IT neurons at their birth dates, using in utero electroporation of green fluorescent protein (GFP) and TdTomato at E12.5 and E14.5, respectively. Comparable survival rates in vitro suggested that reduced ET neuron values reflect altered fate rather than selective death (fig. S3D and Materials and Methods). Consistent with these findings, when we measured cell proliferation in 2D cultures at DIV1 using EdU (Ethynyldeoxyuridine) labeling (Materials and Methods), essentially none of the neurons (identified with TUJ1 staining) were EdU^+^ (4/828 EdU^+^ TUJI^+^). This finding suggests that in vitro neurogenesis, if present at all, accounts for a minority of the neurons in 2D cultures (fig. S3E, left). Supporting this finding, the proportion of in vitro proliferating cells in our transcriptomic dataset was extremely low (5.7% Mki67^+^ cells in glutamatergic class at DIV1; fig. S3E, right).

At the type level, however, many cells were classified as undefined, primarily within the glutamatergic class (Fig. 3, E to G). This reflected a lower molecular definition of cellular identity at the type level, rather than changes in shared transcriptional programs (Fig. 3D). Glutamatergic types showed a lower definition of cellular identity, suggesting that undefined identities result from incomplete acquisition rather than a mixing of identities. Accordingly, expression of type markers was specifically decreased in glutamatergic neurons, while the gene expression of other types appeared less affected (fig. S3F). Consequently, glutamatergic neuron diversity was decreased (Fig. 3H). Non-neuronal cells, on the other hand, maintained their identity definition and diversity (fig. S3F). Whole transcriptome analysis of differentially expressed genes between in vivo and in vitro classes revealed mostly class-specific changes (fig. S3H, left). In glutamatergic neurons, most context-dependent genes coded for proteins associated with synaptic transmission (132 genes), cell-cell communication (81 genes), and ion transport (165 genes) (fig. S3H, right, and table S2). Hence, cell-extrinsic responses are class specific.

To assess maturation pace in vitro, we applied an ordinal regression model trained on temporally dynamic in vivo genes. All classes showed similar maturation delays in vitro (fig. S3I and table S3). This suggests that observed differences in identity definition between classes do not primarily reflect differences in cellular maturity. Similarly, undefined cells within the glutamatergic neuron class had a comparable maturation stage than their defined counterparts (fig. S3J). Together, these results reveal cell type–specific extrinsic controls over glutamatergic neuron identity and diversity that act largely independently of differentiation programs at these developmental stages.

Neural activity plays an important role in neuronal maturation and circuit refinement during development (*20*–*22*). To directly investigate the role of such activity in acquisition of cellular identity and diversity, first, we chronically stimulated 2D cultures with 8 mM KCl [a concentration shown to trigger physiologically relevant depolarization patterns without increasing cell death (*23*, *24*)] and performed snRNA-seq after at DIV17 (fig. S4B). The immediate-early gene *cFos* showed robust up-regulation following this protocol, as confirmed by cFOS immunostaining, indicating effective activation of activity-dependent transcriptional pathways (fig. S4C). At the time of snRNA-seq analysis, however, acquisition of identity was impaired rather than improved compared to nonstimulated conditions (fig. S4, D and E). This suggests that generalized depolarization might not provide the appropriate signals for proper identity acquisition. We hence developed an experimental paradigm that would allow coordinated, rather than random activity patterns. Since L4 neuron molecular identity is particularly sensitive to thalamic input (*5*), we performed in utero electroporation at E14 to target prospective L4 neurons with channel rhodopsin (ChR2), allowing for light-dependent activation of these cells within the 2D culture conditions used above. We used chronic pulses of 20-Hz light stimulation, within the range of thalamocortical afferent firing in vivo (*25*, *26*) between DIV1 and DIV17, and assessed the molecular identity of ChR2-expressing cells using patch sequencing at the end of the stimulation period (fig. S4, G and H).

Minimal transcriptional changes between stimulated and control cells were found (fig. S4I), and all stimulated cells remained undefined (fig. S4J). Thus, chronic optogenetic stimulation in our protocol did not influence acquisition of cell identity. Of note, neither KCl nor Chr2 stimulation increased previously identified activity-dependent genes (fig. S4, I, K, and L) (*27*), likely reflecting the context- and age-specific nature of these transcriptional responses in vivo. Collectively, these findings suggest that neither generalized nor patterned activity is sufficient to drive acquisition of specific cortical cell type identities in vitro, although more comprehensive investigations are needed to fully address the activity’s role in neuronal identity acquisition.

### Canonical circuit connectivity is affected in 2D cultures

To understand the bidirectional relationship between molecular identity and circuit formation in glutamatergic neurons, we next examined connectivity in vivo and in vitro. IT neurons comprise distinct types that assemble to form intracortical circuits: L4 IT neurons receive thalamic input and forward it locally to L2/3 IT neurons, which in turn project to other L2/3 IT neurons. L4 IT to L2/3 IT neuron connectivity is unidirectional: While L4 IT neurons form synapses with L2/3 IT neurons, the reverse is rare. This arrangement ensures the forward flow of sensory information (Fig. 3I, left) (*28*). To assess whether the lack of L4 IT neuron identity [see Figs. 2E and 3, F and G] entailed a corresponding loss in unidirectional L4 IT-to-L2/3 IT connectivity, we targeted these two populations by in utero electroporation at E14 (to target prospective L4 IT neurons) and E15.5 (to target prospective L2/3 IT neurons) (Fig. 3J). To assess L4 IT-to-L2/3 IT connections, L4 IT neurons were targeted with channel rhodopsin (ChR2) at E14, while postsynaptic L2/3 IT neurons were targeted with a reporter gene (TdTomato) at E15.5 [see Materials and Methods and Fig. 3 (I and J)]. Conversely, to assess L2/3 IT-to-L4 IT connections, ChR2 was expressed in L2/3 IT neurons and TdTomato in L4 IT neurons.

We shone blue light to stimulate ChR2-expressing neurons while recording evoked responses in postsynaptic patch-clamped Td-Tomato–expressing neurons. Ex vivo, light stimulation of L4 IT neurons triggered a response in postsynaptic L2/3 IT neurons; L2/3 IT neurons did not evoke responses in L4 IT neurons, consistent with a unidirectional L4-to-L2/3 connectivity (Fig. 3K, top). In vitro, however, a reciprocal connectivity was found between putative L4 IT neurons and L2/3 IT neurons (Fig. 3K, bottom), as present between putative L2/3 neurons in vitro (fig. S4A), matching what is normally found in vivo between L2/3 IT neurons (Fig. 3I, left).

In the mature cortex, L2/3 neurons are highly interconnected with one another [see Fig. 3K and (*29*, *30*)]. The bidirectional connectivity we observe suggests that putative L4 neurons have acquired L2/3-like circuit properties, consistent with their L2/3-like transcriptional signatures revealed by our molecular analysis. Of note, this could, to some extent, reflect developmental immaturity—in an immature state L4 neurons are molecularly L2/3 like—yet there is no evidence that L4 neurons normally receive transient inputs from L2/3 neurons during development.

### Increased cell type–specific glutamatergic neuron identity definition in organotypic cultures

To further explore the influence of cell-extrinsic cues on cell fate and diversity, we transitioned to organotypic slice cultures. These slices offer a more complex extracellular environment compared to 2D cultures while maintaining the accessibility of an in vitro system.

**Fig. 4.**
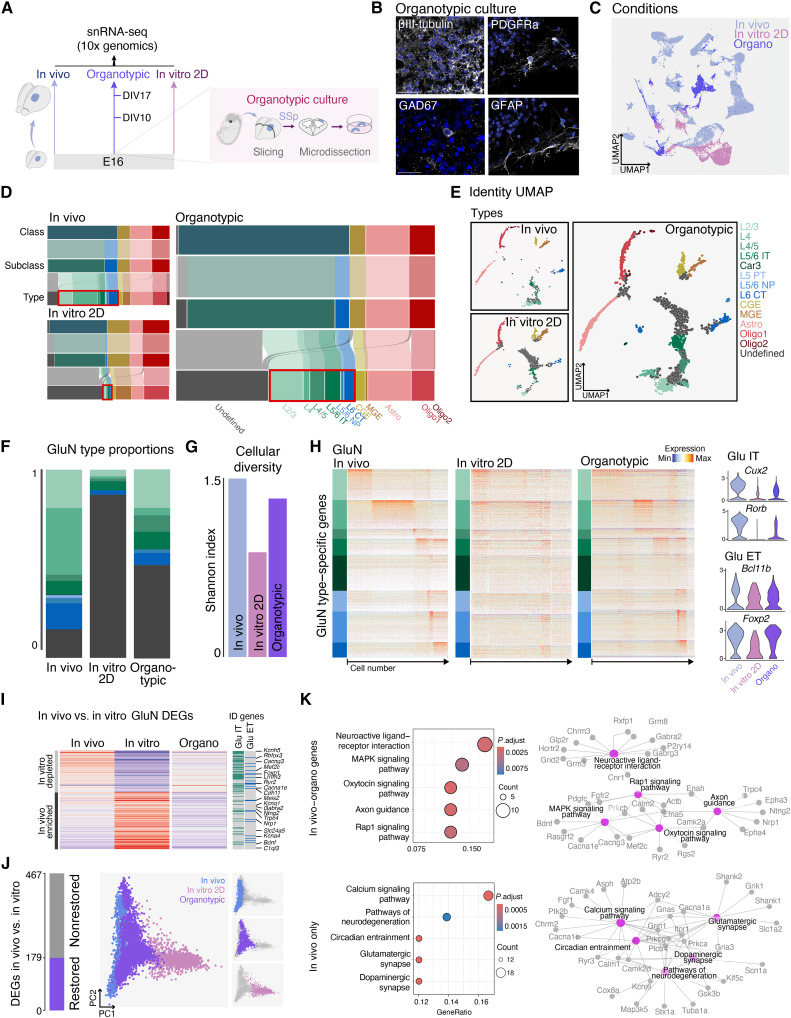
Organotypic culture partially restores glutamatergic neuron type identity. (**A**) Schematic of the experimental pipeline. (**B**) Immunofluorescence of DIV17 organotypic cultures for glutamatergic neurons (βIII-tubulin), GABAergic neurons (GAD67), oligodendrocyte precursors (PDGFRα), and astrocytes (GFAP). Scale bar, 50 μm. (**C**) UMAP of snRNA-seq dataset including in vivo, 2D culture (data of Fig. 3) and organotypic culture (new). (**D**) Sankey diagrams for all populations and cumulative proportions for glutamatergic types (same data of Fig. 3 for in vivo and 2D cultures). (**E**) Identity UMAPs for cell types are displayed for in vivo and 2D cultures (data of Fig. 3) and organotypic culture. Gray: undefined types. (**F**) Proportions of glutamatergic types are displayed for in vivo and 2D (data of Fig. 3) and organotypic culture. Gray: undefined types. (**G**) Shannon index for glutamatergic neurons across the three conditions. (**H**) Identity heatmaps of glutamatergic neuron for in vivo and in vitro (data of Fig. 3) and organotypic culture. Right: Expression levels of example genes. (**I**) Top: Heatmap showing the average expression of in vivo versus in vitro DEGs (rows), restored in organotypic culture glutamatergic neurons. (P14 for in vivo and DIV17 for 2D and organotypic). Right: IT/ET neurons ID genes. (**J**) Principal components analysis (PCA) of average expression of the restored genes in organotypic culture for each cell (dots), color-coded per condition. Right: Panels show single conditions. (**K**) KEGG pathway analysis of genes whose expression was restored in organotypic slices glutamatergic neurons (left). Cnet plot indicating that the genes involved in the top five pathways are reported (right).

We hypothesized that this could mitigate some of the defects observed above. To test this, we cultured 300-μm-thick coronal slices of E16 embryonic somatosensory cortex, using the same medium as before (Fig. 4, A and B, and Materials and Methods). We performed snRNA-seq at DIV10 and DIV17 (Fig. 4C) and analyzed identity and diversity at each level of the cellular hierarchy. At the class and subclass levels, the distribution of cellular populations in organotypic cultures more closely resembled in vivo distributions, contrasting with observations in 2D cultures. This included ET neuron proportions closer to the in vivo condition. A larger fraction of glutamatergic neurons acquired type-specific identities, although not fully matching in vivo levels (Fig. 4, D to F, and fig. S5, A to C). Correspondingly, glutamatergic neuron type diversity approached in vivo values (Fig. 4G). The molecular definition of glutamatergic neuron identity was higher than in 2D cultures (Fig. 4H, left). This was evident in the expression of key transcripts: IT neuron markers *Cux2* and *Rorb* (*31*, *32*) and ET neuron markers *Bcl11b* and *Foxp2* (*33*, *34*) were more highly expressed in organotypic cultures compared to 2D cultures (Fig. 4H, right). More broadly, the average expression level of cell type–specific markers increased to levels observed in vivo for almost all cell populations (fig. S5D).

To assess whether the increased molecular definition of cellular identity in organotypic conditions reflected effects on cellular maturation, we performed pseudo-time analysis (fig. S5E). This analysis revealed comparable maturity levels in both 2D cultures and organotypic cultures. This suggests that the enhanced molecular definition of cellular identity observed in organotypic cultures is largely independent of overall maturation levels.

To identify the transcriptional programs at play in preserving in vivo–like glutamatergic neuron type–specific identities, we identified differentially expressed genes between in vivo and 2D-cultured neurons at P14 and DIV17 (467 genes). This analysis revealed a subset of genes (179) with similar patterns of expression in vivo and in organotypic slices, compared to 2D cultures, i.e., “in vivo + organo” genes (Fig. 4I and fig. S5F). Of all the genes that were different, 38% were “restored.” Their average expression in organotypic cultures closely recapitulates the expression pattern of in vivo cells, as revealed by principal components analysis (Fig. 4J and fig. S5G).These genes included IT and ET type–specific markers (e.g., *Rbfox3* and *Foxp1*; Fig. 4I, right) and genes involved in extrinsic regulation, such as those associated with neuroactive ligand-receptor interaction, axon guidance, and mitogen-activated protein kinase (MAPK) signaling pathways (e.g., *Epha3* and *Nrp1*; Fig. 4K, top), as identified by Kyoto Encyclopedia of Genes and Genomes (KEGG) pathway analysis. Notably, the MAPK signaling pathway, which is also enriched in VB^−^ model DEGs (differentially expressed genes) (fig S5H, bottom), plays crucial roles in cortical development through extrinsic modulation, and its dysregulation has been linked to neurodevelopmental conditions such as autism spectrum disorder (*35*, *36*). To gain deeper insight into these preserved genes, we performed literature-based annotation of the top 50 in vivo + organo genes. This analysis revealed that 34 genes had functions in neuronal maturation, synaptic development, and activity-dependent plasticity (table S5). Examples include *Rbfox3* (NeuN) for neuronal differentiation, *Bndf* for synaptogenesis, and *Rorb* for layer 4 identity. Several are activity dependent (e.g., *Nnat* and *Kcnh5*), including an activity-dependent epigenetic regulator (*Hdac9*), and related to axonal projection (e.g., *Crim1* and *Efna5*), consistent with their down-regulation in 2D cultures leading to immature transcriptional signatures and altered connectivity. While the preserved genes were mostly linked to axon guidance and elongation, the genes that were not preserved in either of in vitro models were linked to later developmental events, including synaptogenesis (Fig. 4K, bottom, “in vivo–only” genes). Comparing these molecular pathways with those affected in Reeler and VB^−^ mice revealed that different environmental perturbations (altered positioning, loss of thalamic input, or absence of tissue architecture) affect largely distinct transcriptional programs rather than overlapping pathways (fig. S5H and table S6), indicating condition-specific molecular responses to environmental disruption. These findings suggest that organotypic cultures preserve key extrinsic cues necessary for establishing and maintaining cell type–specific molecular identities but do not fully recapitulate the necessary conditions for maturation.

## DISCUSSION

Our data address cell type–specific extrinsic influences on neuronal fate. Using a bioinformatic framework grounded in population-specific cellular identity, we demonstrate the remarkable robustness of class and subclass identities, which remain conserved even in 2D dissociated cultures. This bioinformatic approach for cell classification facilitates comparisons of cellular diversity across conditions and could be extended beyond the current study to compare cellular identity across model systems.

Our findings suggest that differences in maturation rates across cell types may contribute to cell type–specific temporal windows of plasticity. Further studies should address limitations in our taxonomic resolution, which is higher for glutamatergic neurons (eight types) than for other populations (two types for GABAergic neurons and oligodendrocytes and one for astrocytes). This difference may influence apparent maturation timelines. In addition, GABAergic neurons are already lineage-specified (medial ganglionic eminence/caudal ganglionic eminence) before reaching the cortex, while glutamatergic differentiation occurs within our sampling window, creating an uneven baseline for cross-population comparisons. Hence, taxonomic resolution and developmental starting points should be considered when comparing maturation rates across neural cell populations.

In vivo, acquisition of cell type identity was only minimally affected by changes in cellular position (Reeler mice) or input (VB^−^ mice). In Reeler mice, however, the distribution of cells across canonical types was affected, as evidenced by an increase in L2/3 IT neurons at the expense of L4 IT neurons. This could reflect an incomplete maturation of L4 IT neurons, which initially send long-range axons to the opposite hemisphere (*16*). Since L4 IT identity is not fully defined before P14, these neurons may thus initially have a L2/3 IT-type molecular identity that normally matures to become L4 IT in an input-dependent manner.

While this study sheds light on the influence of extrinsic factors on neuronal cell fate acquisition, the models used have inherent limitations. The Reeler cortex exhibits disruptions beyond simple dyslamination, including altered morphology and input that likely influence our observations independent of strictly positional effects (*12*). Similarly, the loss of somatosensory input in VB^−^ mice likely triggers cascading changes in intracortical connectivity and activity patterns throughout development (*5*, *16*), such that observed effects cannot be reduced to the primary phenotype of these mutants.

Our in vitro experiments reveal a neuron class–specific susceptibility to extrinsic cues for identity acquisition, with glutamatergic neurons being more susceptible than GABAergic neurons. This difference may reflect the resolution thresholds used to define cell types in this study, as some subtypes of GABAergic neurons (not studied specifically here) have been shown to mature differently in different environments (*37*, *38*) and respond to changes in the microcircuitry (*39*). Similarly, astrocytes change their properties in vitro (*40*). This is reflected in our 2D cultures by an increase in the number of differentially expressed genes in vitro. Last, oligodendrocyte maturation highly depends on culture conditions (*37*, *38*, *41*, *42*) and respond to changes in the microcircuitry (*39*). Similarly, astrocytes change their properties in vitro (*40*). This is reflected in our 2D cultures by an increase in the number of differentially expressed genes in vitro. Last, oligodendrocyte maturation highly depends on culture conditions (*41*, *42*), which is reflected here by a specific decrease of the mature oligodendrocytes type (Oligo2).

The improved molecular definition of cellular identity in organotypic slice cultures underscores the crucial role of extracellular context in determining neuronal fate. This finding supports the significance of extrinsic modulation in cell differentiation and highlights the potential of advanced 3D culture systems, such as organoids, in replicating complex neural tissues.

While the importance of environmental conditions for neuronal maturation has been previously identified [see, for e.g., (*43*)], our study takes a distinct approach by examining cells born in vivo and directly comparing the same cellular populations from a common starting point across different environmental conditions. This design isolates specific effects on neuronal maturation, rather than confounding effects on neuronal generation from progenitors. Our work also complements transplantation studies, for which our molecular characterization provides a framework to understand how host environmental cues are critical for proper neuronal integration and function [see e.g., (*44*, *45*)]. Last, further investigation into these cell-extrinsic factor–dependent mechanisms, including the role of activity in identity acquisition, may identify novel therapeutic targets in neurodevelopmental disorders, particularly those influenced by gene-environment interactions.

## MATERIALS AND METHODS

### Experimental models

All experimental procedures were approved by the Geneva Cantonal Veterinary Authority. E0.5 was established as the day of vaginal plug. Wild-type CD1 mice were provided by Charles River Laboratories.

Male and female embryos between E12.5 and E15.5 were used for the in utero electroporation experiments and mice between E16 and P21 for the postnatal experiments. Pregnant dams were kept in single cages, and pups were kept with their mothers until P21, in the institutional animal facility under standard 12:12-hour light/dark cycles.

Reeler mice (https://jax.org/strain/000235) were obtained from J. F. Staiger (University Medical Center Göttingen, Centre for Anatomy, Institute of Neuroanatomy, Kreuzbergring 36, D-37075, Göttingen). Mutant mice were obtained by crossing Reln^rl/+^ heterozygous mice. The line was genotyped using the following primers: CTGTCCTCACTCTGCCCTCTGGTAG, CTACACAGTTGACATACCTTAATCTAC, and ACTTGCATTAATGTGCAGTGTTGTC.

A VB^−^ mouse model was obtained by crossing Slc6a4-Cre transgenic mice (*46*) with ROSA-DTA mice (RRID:IMSR_JAX:009669) as in (*5*). The lines were genotyped using the following primers: Cre Forward: ATT TGC CTG CAT TAC CGG TCG; Cre Reverse: CCC CAG AAA TGC CAG ATT ACG TAT ATC; R26 DTA Forward: CAT CAA GGA AAC CCT GGA CTA CTG; R26 WT Forward: AAA GTC GCT CTG AGT TGT TAT; R26 Reverse: GGA GCG GGA GAA ATG GAT ATG.

### Embryonic cortical 2D primary cultures

Somatosensory cortices from E16 mouse embryos were microdissected in 1× ice-cold Hanks’ balanced salt solution (HBSS) (Gibco, catalog no. 14025092), mechanically dissociated with 1× TrypLE Express Enzyme (Gibco, catalog no. 12604013) after 5-min incubation at 37°C, and collected by centrifugation (500*g* for 5 min, 4°C). Dissociated cells were then filtered through a 70-μm cell strainer (VWR, 352350) and plated in 24-well plates containing neurobasal medium presupplemented with penicillin-streptomycin (Thermo Fisher Scientific, catalog no. 15070063), 2% B27 supplement, and 0.5 mM glutamine onto glass coverslips precoated overnight at 37°C with poly-d-lysine (0.1 mg/ml; Advanced Biomatrix, catalog no. 5174-5MG) and laminin (0.01 mg/ml; Thermo Fisher, catalog no. 23017015). Cultures were kept in incubators at 37°C with 5% CO_2_, and partial replacement of the culture media was done every 2 to 3 days.

For EdU labeling, cells were exposed to 20 μM EdU for 2 hours on the day of plating (DIV0) and analyzed the day after (DIV1). EdU-labeled human embryonic kidney 293T cells were used as positive control. The EdU signal was revealed using the Click-iT EdU Cell Proliferation Kit with Alexa Fluor 647 dye (Thermo Fisher Scientific, catalog no. C10340).

### Embryonic cortical organotypic cultures

Somatosensory cortices from E16 mouse embryos were microdissected in 1× ice-cold HBSS (Gibco, catalog no.14025092), and acute slices were obtained using Leica Vibratome VT1220S (cutting parameters: 0.1-mm/s speed, 1-mm amplitude, 300-μm thickness). Microdissected S1 cortices were subsequently cultured with the same medium of 2D cultures using organotypic cell culture inserts (Merck Millipore, PICM03050). Cultures were kept in incubators at 37°C with 5% CO_2_, and full media change was done once every 2 to 3 days.

### In utero electroporation and CFSE injection

Timed pregnant CD1 mice were preemptively injected with 300 μl of buprenorphine (0.1 mg/kg; Temgesic, Benckiser, Switzerland) in 0.9% NaCl and subsequently anaesthetized with isoflurane (5% induction, 3% during the surgery). Embryos were injected unilaterally with 700 nl of plasmid solution, diluted in endotoxin-free TE (Tris-EDTA) buffer and 0.002% Fast green FCF (Sigma-Aldrich), in the lateral ventricle. Embryos were electroporated by placing the head between circular tweezer electrodes (5 mm in diameter; Sonidel Limited, UK), while five electric pulses (30 V for E12.5, 40 V for E14.0, 50 V for E15.5, 50 ms at 1 Hz) were delivered with a square-wave electroporator (Nepa Gene, Sonidel Limited, UK). For Flashtag labeling, embryos were injected unilaterally with 700 nl of 10 mM carboxyfluorescein succinimidyl ester (CellTrace CFSE, Life Technologies, C34554), diluted in dimethyl sulfoxide and 0.002% Fast green FCF (Sigma-Aldrich), in the lateral ventricle.

### Plasmids

The following plasmids were used for in utero electroporations: pAAV-CAG-tdTomato (Addgene, #83029), pCAG-GFP (Addgene, #16664), pAAV-CAG-ChR2(E123T;T159C)-2A-tDimer (Addgene, #85399), and pCAG-ChR2-Venus (Addgene, #15753).

### KCl stimulation

#### Validation of KCl stimulation

Acute (1 hour) 8 mM treatment was performed at DIV3 primary cultures. Activation of the cells was monitored by measuring cFOS expression by immunofluorescent staining.

#### KCl stimulation for single-nuclei sequencing

Primary somatosensory cultures were cultured with neurobasal culture medium containing 8 mM KCl until nuclei collection at DIV17.

### Chronic optogenetic stimulation

For chronic optogenetic stimulation of primary cultures, cultures were prepared from E16 brains electroporated at E12.5 with pCAG-tdTomato plasmid or E14 with pCAG-tdTomato plasmid alone or with a mix of pCAG-tdTomato plasmid and pAAV CAG ChR2 E123T T159C 2A tDimer plasmid. Starting at DIV1, cultures were chronically optogenetically stimulated by applying a protocol of train stimulation of blue light-emitting diode (train of 20 stimulations; 10-ms stimulation duration; 50-ms stimulation interval; 30-s intertrain interval) until patch sequencing at DIV17.

### Single-nuclei RNA sequencing

#### SnRNA-seq of mouse somatosensory cortices

Somatosensory cortices were collected from E17 embryos and P1, P7, and P14 CD1 mice. For the Reeler model, at P23, three brains were collected from three independent litters of Reln^rl+/+^ mutants and corresponding Reln^rl+/−^ control littermate. VB^−^ somatosensory cortices were collected from three Slc6a4-Cre;LSL-DTA mutant mice and from three LSL-DTA^+/−^ control mice at P14 from the same litter. For E17 time point, somatosensory cortices from five embryos were microdissected in 1× ice-cold HBSS (Gibco, catalog no. 14025092). For postnatal time points, 300- to 400-μm-thick coronal slices were prepared, and primary somatosensory cortices were microdissected. The tissue was preserved at −80°C until use for the single-nuclei suspension, which was obtained through douncer homogenization (Sigma-Aldrich, D8938) using an adaptation of the protocol for the Nuclei EZ buffer kit (Sigma-Aldrich, NUC101). For dissociations, after being thawed on ice, the tissue was resuspended in 2 ml of ice-cold extraction buffer (Nuclei EZ prep buffer) and dounce-homogenized on ice until disintegration (15 to 25 pestle A and B). After incubation for 5 min in 4 ml of EZ buffer total, the extracted nuclei were collected by centrifugation (500*g* at 4°C, 5 min). The extraction procedure was repeated once, before performing a washing step, in which nuclei were resuspended in washing buffer composed of 1% bovine serum albumin (BSA) in phosphate-buffered saline (PBS), with SuperASE-In (50 U/ml; Thermo Fisher Scientific, catalog no. AM2696) and RNasin (50 U/ml; Promega, catalog no. N2611), after centrifugation. Last, the nuclei were resuspended in 1 ml of washing buffer and filtered through a 30-μm strainer into a new low-binding 1.5-ml tube and processed for fluorescence-activated nuclei sorting (FANS) on Beckman Coulter MoFlo Astrios, after incubation with Hoechst (1:500; Invitrogen, H3570) for 5 to 10 min on ice. A total of 20,000 nuclei were sorted, and 42 μl of nuclei suspension was used to load the Chromium Next GEM Single Cell 3’ Kit v2 (10x Genomics), according to the manufacturer’s instructions. Each library contained nuclei from only one time point.

#### snRNA-seq from primary cultures

Single-nuclei suspensions were obtained as indicated in the manual of the Nuclei EZ prep (catalog no. NUC-101), and nuclei isolation was performed through FANS as indicated for in vivo preparations. snRNA-seq preparations were performed as indicated in manufacturers’ instructions.

#### snRNA-seq from organotypic cultures

Nuclei were harvested from cortical organotypic slices by douncer homogenization (pestle A seven times and pestle B six times) by Nuclei EZ buffer extraction, similarly to tissue preparations. Nuclei were isolated through FANS, and snRNA-seq preparations were performed according to manufacturers’ instructions.

#### Analysis of snRNA-seq data

All single-nucleus transcriptomics data generated with 10x Genomics technology were mapped, and count matrix was generated using 10x Genomics cellranger program (v6.1.1). The patch sequencing data were mapped using STAR (v2.7.2b), and count matrices were generated in R using the rtracklayer package (v1.62.0).The count matrices were then analyzed using the R statistical Software (v4.1.1), Seurat package (v5.0.1), ggplot (v3.3.6), DoubletFinder (v2.0.3), clusterProfiler (v4.10.1), bmrm (v4.4), vegan (v2.6-6.1), and bayestestR (v0.13.2).

##### Cell filtering and quality control

The data of CD1 mice in vivo and in vitro, with KCl treatment and organotypic slice cultures; transcriptomes were considered as single nucleus when expressing more than 500 genes, showing less than 30,000 UMI (unique molecular identifier) counts, which the UMI/gene ratio was superior at 1.2 and percent of mitochondrial RNA lower than 2%. The Reeler mutant and control P23 transcriptomes were filtered for expressing 1000 genes and more, UMI counts between 2500 and 15,000 and a percent of mitochondrial RNA lower than 1%. The VB mutant and control transcriptomes were filtered for expressing more than 500 genes, expressing less than 20,000 UMIs and less than 2% of mitochondrial RNA. In addition to quality controls, cells annotated as microglia, endothelial, and vascular-related identities were excluded from further analysis for all datasets. For patched-sequenced nuclei, 4 of 48 nuclei were excluded, because of low number of detected genes (3 nuclei) or low proportion of mapping reads (1 nucleus). The number of nuclei analyzed for each condition is reported in table S4.

##### Cell population hierarchy

Public single-cell transcriptomic dataset of the entire adult mouse neocortex and hippocampus (*13*) was used to build the hierarchy of cell populations. Cells harvested from the somatosensory primary cortex were isolated, and endothelial and vascular cell types (endo, micro-PVM, SMC-peri, and VLMC) along with Cajal-Retzius cells were removed from further analysis. Cells were then analyzed following the Seurat standard procedure. The data were clustered using the FindClusters() function from Seurat with different values of the resolution parameter corresponding to 0.0005 for cell classes, 0.001 for subclasses, and 0.08 for types (fig. S1A).

##### Selection of population marker genes

The three reference datasets (CD1 P14, DTA^flox/flox^ P14, and Reeler^+/−^ P23) were merged and integrated using CCA integration from Seurat v5 pipeline. Following integration, cells were annotated to cell types, subclasses, and classes using a deep neural network trained to classify cells based on the Yao *et al.* (*13*) dataset as described previously (*47*). The code and details of the DNN (deep neuronal network) training are available at https://github.com/baumannnatalia/Celltype_Classifier_DNN. The marker genes were identified using the FindMarkers() function from Seurat, with minimal log_2_ fold change value of 0.6 and minimal difference in percentage of expressing cells of 0.1.

##### Identity UMAP generation

All count matrices were scaled using the SCTransform() function from the Seurat package. The average gene expression of the different population markers was measured as the mean of scaled expression of all genes of a given population. The averaged population gene expression was then used for UMAP visualization. All reference dataset cells used for marker identification were used as reference. For each cell, the 10 nearest reference neighbors were identified using Euclidean distance on the UMAP 2D space. The class, subclass, and type identities of the nearest neighbors were evaluated to assign a cell to a certain population. If 9 or 10 neighbors belonged to the same population, then the cell was annotated to this population. If the neighbors’ identities were mixed (less than nine neighbors within the same population), then the cell was considered undefined. Each hierarchical level was annotated independently, meaning that a cell could be assigned to a population for higher hierarchical level but not for the lower ones (Fig. 1D).

##### Differentially expressed genes, gene ontologies, and KEGG pathway enrichment

The differentially expressed genes shown in fig. S3 were measured using the FindMarkers() function from the Seurat package. The genes were filtered to have a log fold change higher than 0.6, an adjusted *P* value lower than 0.05, and a difference in percentage of expressing cells higher than 0.2. The enrichment analysis of gene ontologies within differentially expressed genes was done using the enrichGO function for biological processes from the clusterProfiler package. The 10 most enriched gene ontologies were grouped into larger function classes annotated by hand and shown on fig. S3. The KEGG pathway enrichment was performed with enrichKEGG from the clusterProfiler package. The top 10 enriched pathways are shown in the dot plot and the top 5 in the network of Fig. 4K.

Mann-Whitney tests were used to compute *P* values for gene expression differences.

Figure 2H: L4 expression L4 cells: control versus Reeler, *P* = 2.8^−100^; control versus VB^−^, *P* = 8.9^−50^; L2/3 cells: control versus Reeler, *P* = 2.6^−22^; control versus VB^−^, *P* = 2.6^−5^. Figure S2G: Control versus Reeler L2/3, *P* = 1; L4, *P* = 1.43^−11^; L4/5 IT, *P* = 1; L5/6 IT, *P* = 1; Car3, *P* = 0.98; L5 PT, *P* = 1; L5/6 NP (near projecting), *P* = 1; layer 6 corticothalamic neurons (L6 CT), *P* = 1; undefined, *P* = 5.68^−18^. Control versus VB^−^ L2/3, *P* = 1.06^−10^; L4, *P* = 2.50^−77^; L4/5 IT, *P* = 0.12; L5/6 IT, *P* = 1; Car3, *P* = 5.53^−9^; L5 PT, *P* = 1; L5/6 NP, *P* = 1; L6 CT, *P* = 7.25^−9^; undefined, *P* = 3.99^−65^. Figure S3F in vivo versus in vitro L2/3, *P* = 2.2^−37^; L4, *P* = 5.46^−105^; L4/5 IT, *P* = 2.99^−2^; L5/6 IT, *P* = 0.55; Car3, *P* = 1; L5 PT, *P* = 6.12^−4^; L5/6 NP, *P* = 3.16^−7^; L6 CT, *P* = 4.43^−24^; undefined, *P* = 0.00. Figure S5C in vivo versus organotypic L2/3, *P* = 1; L4 *P* = 1.16^−214^; L4/5 IT, *P* = 2.83^−14^; L5/6 IT, *P* = 8.7^−2^; Car3, *P* = 1; L5 PT, *P* = 1.11^−11^; L5/6 NP, *P* = 0.12; L6 CT, *P* = 2.97^−19^; undefined, *P* = 2.44^−149^. Organotypic versus in vitro L2/3, *P* = 3.00^−39^; L4, *P* = 9.58^−5^; L4/5 IT, *P* = 2.77^−15^; L5/6 IT, *P* = 1.63^−5^; Car3, *P* = 1; L5 PT, *P* = 1; L5/6 NP, *P* = 1.13^−5^; L6 CT, *P* = 2.25^−6^; undefined, *P* = 4.2^−106^.

##### Pseudo-age prediction

Pseudo-age was built using ordinal regression models from bmrm package as previously described (*48*). Briefly, a regularized ordinal regression was used for order of cells harvested at different time points from the in vivo dataset (E17, P1, P7, and P14). During modeling, a weight is assigned to each gene to optimize the correct ordering of cells. To avoid overfitting, a cross-validation was performed to assess the performance of the modeling. To retrieve the optimal number of genes for the pseudo-age model, different numbers of genes were tested. The efficiency of the models was assessed by measuring the overlap of distributions of the cross-validation values of cells harvested at different time points, using the Overlap function from the bayestestR package. A high overlap coefficient means poor separation of cells from different time points, and we selected the minimal number of genes for which the overlap coefficient was minimal, here 180 genes (figs. S3, H and I, and S4E). The pseudo-age value of in vivo cells used for model training corresponds to their cross-validation value, while for the 2D in vitro and organotypic cells, the prediction values are shown.

##### Shannon diversity index

To assess the diversity of cell population from each class and in each extrinsic modulation condition, all data from in vivo E17 to P14, in vitro DIV1 to DIV17, and organotypic DIV10 and DIV17 were merged (without integration). A clustering analysis was performed using the FindClusters function from the Seurat package with a resolution parameter set to 1. The Shannon diversity index was measured from the number of cells within each cluster using the diversity function from the vegan package with the index option set to “shannon.”

##### Sensory activity gene expression

The genes from Hrvatin *et al.* (*27*) were provided in a table indicating regulation of gene expressions early (1 hour) and late (4 hours) following sensory stimulation in each cell type. We retrieved a cell class–specific list of activity-regulated genes by retrieving only those reported in a specific cell class (e.g., a gene X down-regulated upon activity in L4 neurons belongs to down-regulated genes for glutamatergic cells). The results shown in fig. S4K are the average expression of the cell class–specific genes. The differential expression analysis performed for the volcano plots in fig. S4 (F and K) was performed using the FindMarkers function of the Seurat package with default parameters.

### Immunofluorescence and imaging

#### Immunofluorescence staining of 2D primary culture

Coverslips were fixed with 4% paraformaldehyde (PFA) in PBS for 15 min at room temperature and washed three times in PBS. Blocking and permeabilization were performed by incubating the cultures, at room temperature, with 1% BSA, 0.5% Triton X-100 in PBS for 45 min. Afterward, incubation with the primary antibodies was done overnight at 4°C in antibody solution (0.5% BSA and 0.1% Triton X-100 in PBS). Following three washes in PBS, the coverslips were incubated at room temperature for 1 hour with secondary antibody mix and Hoechst and lastly mounted on glass slides, after being washed three times in PBS. Images were acquired on Nikon A1r or LSM800 confocal microscopes (Carl Zeiss).

#### Immunofluorescence staining of organotypic culture

Slices were fixed with 4% PFA in PBS for 30 min at room temperature and washed three times in PBS. Blocking and permeabilization were performed by incubating the cultures, at room temperature, with 1% BSA and 0.5% Triton X-100 in PBS for 45 min. Afterward, incubation with the primary antibodies was done overnight at 4°C in antibody solution (0.5% BSA and 0.1% Triton X-100 in PBS). Following three washes in PBS, the coverslips were incubated at room temperature for 1 hour with secondary antibody mix and Hoechst and lastly mounted on glass slides, after being washed three times in PBS. Images were acquired on Nikon A1r or LSM800 confocal microscopes (Carl Zeiss).

The following antibodies were used: chicken anti-MAP2 (1:5000; Abcam, ab5392); goat anti-PDGFRa (1:500; Bio-Techne, AF1062); rabbit anti–glial fibrillary acidic protein (GFAP; 1:1000; Abcam, ab7260); rabbit anti-DCX (1:500; Cell Signaling, 4604); mouse anti-GAD67 (1:500; Merck Millipore, MAB5406); rabbit anti–β3 tubulin (1:1000; BioLegend, PRB-435P); and rabbit anti-cFOS (1:1000; Synaptic System, 226008).

#### Electrophysiology, optogenetics, and patch sequencing

For ex vivo patch-clamp connectivity recordings, 300-μm-thick coronal slices were prepared from P14 to P21 CD1 mice electroporated at E14 or E15.5 with pCAG-Venus-Chr2 plasmid. Slices were kept for 30 min in artificial cerebrospinal fluid (ACSF) at 33°C (125 mM NaCl, 2.5 mM KCl, 1 mM MgCl_2_, 2.5 mM CaCl_2_, 1.25 mM NaH_2_PO_4_, 26 mM NaHCO_3_, and 11 mM glucose, saturated with 95% O_2_ and 5% CO_2_) before recording. The slices were then transferred in the recording chamber, submerged, and continuously perfused with ACSF.

For in vitro patch-clamp connectivity recordings, recordings were performed on cocultured in utero-electroporated neurons at E14 with pCAG-Venus-ChR2 plasmid and electroporated at E15.5 with pCAG-tdTomato plasmid or in utero-electroporated at E15.5 with pCAG-Venus-Chr2 plasmid and electroporated at E14 with pCAG-tdTomato plasmid. For E15 to E15 connectivity versus E15 to E14 born neuron connectivity, patch-clamp recordings were performed on cocultured in utero-electroporated neurons at E15.5 with pCAG-Venus-Chr2 plasmid, in utero-electroporated neurons at E15.5 with pCAG-tdTomato plasmid, and E14.5 Flashtag-labeled neurons. Labeled neurons were fluorescence-activated cell sorting (FACS)–sorted at E16, cocultured, and patched at DIV17. Under this triple culture condition, at DIV17, CFSE has been degraded and is no longer visible in the cells; therefore, nonfluorescent cells were considered as being E15 Flashtag-labeled neurons. The internal solution used for the experiments contained 140 mM potassium methansulfonate, 2 mM MgCl_2_, 4 mM NaCl, 0.2 mM EGTA, 10 mM Hepes, 3 mM Na_2_-adenosine triphosphate, 0.33 mM guanosine triphosphate, and 5 mM creatine phosphate (pH 7.2, 295 mOsm). EPSC were evoked in the presence of 1 μM tetrodotoxin (Tocris, CAS number 18660-81-6) and 1 mM 4-aminopyridin (Hellobio, CAS number 504-24-5) to avoid polysynaptic recording and 10 μM bicuculine (Tocris, CAS number 485-49-4) by a 10-ms blue-light pulse at 0.1 Hz delivered through the 60× objective. For resting membrane potential and excitability recordings, immediately after the whole-cell configuration, the neuron was placed in current clamp mode, and four steps of 50, 100, 200, and 400 pA were applied for 500 ms. Membrane potential was monitored every 10 s and averaged for five consecutive acquisitions, within the first 2 min after the whole-cell configuration establishment. Access resistance was monitored by a hyperpolarizing step of 14 mV at each sweep, every 10 s. Liquid junction potential was not corrected. Recordings were amplified (Multiclamp 700, Axon Instruments), filtered at 5 kHz, digitalized at 20 kHz (National Instrument Board PCI-MIO-16E4, IGOR WaveMetrics), and stored on a personal computer for further analyses (IGOR PRO WaveMetrics). Statistical analyses (fig. S3A) were performed using the Kruskal-Wallis test for multiple comparisons or unpaired *t* test in the case of two condition comparisons. *P* values Vm: P7 versus P14: >0.9999, P7 versus DIV10: 0.0005, P7 versus DIV17: 0.0076, P14 versus DIV10: 0.037, P14 versus DIV17: 0.1329, and DIV10 versus DIV17: >0.9999. *P* values number of action potentials: P14 versus DIV17: 0.0144, P14 versus P1: <0.0001, DIV17 versus P1: 0.0018. *P < 0.05, **P < 0.01, ***P < 0.001, ****P < 0.0001, and not significant (ns).

For collection of cells for patch single-cell RNA sequencing, DIV17 neurons electroporated at E12.5 with pCAG-tdTomato plasmid or E14 with pCAG-tdTomato plasmid alone or with a mix of pCAG-tdTomato plasmid and pAAV CAG ChR2 E123T T159C 2A tDimer plasmid were patched with a patch pipette containing 1 μl of internal solution supplemented with ribonuclease (RNase) inhibitor (0.4 U μl^−1^; Takara). To facilitate the aspiration of the cell, low pipette resistance was used (4 to 3 megohm). Once in whole-cell configuration, the neuron was placed in current clamp mode, and four steps of 50, 100, 200, and 400 pA were applied for 500 ms. Following this, the recording was switched to voltage clamp mode with the membrane potential clamped at −60 mV. A slit depression was applied to the pipette to aspirate the intracellular content. The complete aspiration of the cell was observed under high magnification (Nikon Eclipse FN1, 60× lens) as retraction of the cytoplasm and total aspiration of the nucleus. The patch pipette was then slowly retracted, and the pipette tip containing the cell content was broken into a polymerase chain reaction RNase-free Eppendorf containing 8 μl of lysis buffer from the SMART-Seq v4 3′ DE Kit and stored at −80°C until further processing.

##### Single-cell RNA sequencing of patched cells

cDNA synthesis and preamplification were performed using the SMART-Seq v4 3′ DE Kit following the manufacturer’s instructions (Takara). Single-cell RNA sequencing libraries of the cDNA were prepared using the Nextera XT DNA library prep kit (Illumina). Libraries were multiplexed and sequenced according to the manufacturer’s recommendations with paired end reads using the HiSeq4000 platform (Illumina) with an expected depth of one million reads per single cell and a final mapping read length of 50 bp. Each pool contained cells from different collection days and conditions. All single-cell RNA capture and sequencing experiments were performed at the Genomics Core Facility of the University of Geneva. The sequenced reads were aligned to the mouse genome (GRCm38) using the STAR aligner. The number of reads per transcript was calculated with the open-source HTSeq Python library. All analyses were computed on the Vital-It cluster administered by the Swiss Institute of Bioinformatics. Cells expressing <2000 genes or >12% of mitochondrial genes were excluded.
