# Supplementary material for: Cell-extrinsic controls over neocortical neuron fate and diversity
Source: Sci Adv. 2025 Sep 17;11(38):eadw0218. doi: 10.1126/sciadv.adw0218 (PMC12442843; doi:10.1126/sciadv.adw0218)
Supplement: Supplementary file 1 — Figs. S1 to S5 Legends for tables S1 to S6 [file sciadv.adw0218_sm.pdf]

Science Advances
AAAS

Supplementary Materials for

Cell-extrinsic controls over neocortical neuron fate and diversity

Natalia Baumann *et al.*

Corresponding author: Sabine Fièvre, sabine.fievre@unige.ch; Denis Jabaudon, denis.jabaudon@unige.ch

Sci. Adv. **11**, eadw0218 (2025)
DOI: 10.1126/sciadv.adw0218

The PDF file includes:

Figs. S1 to S5
Legends for tables S1 to S6

Other Supplementary Material for this manuscript includes the following:

Tables S1 to S6

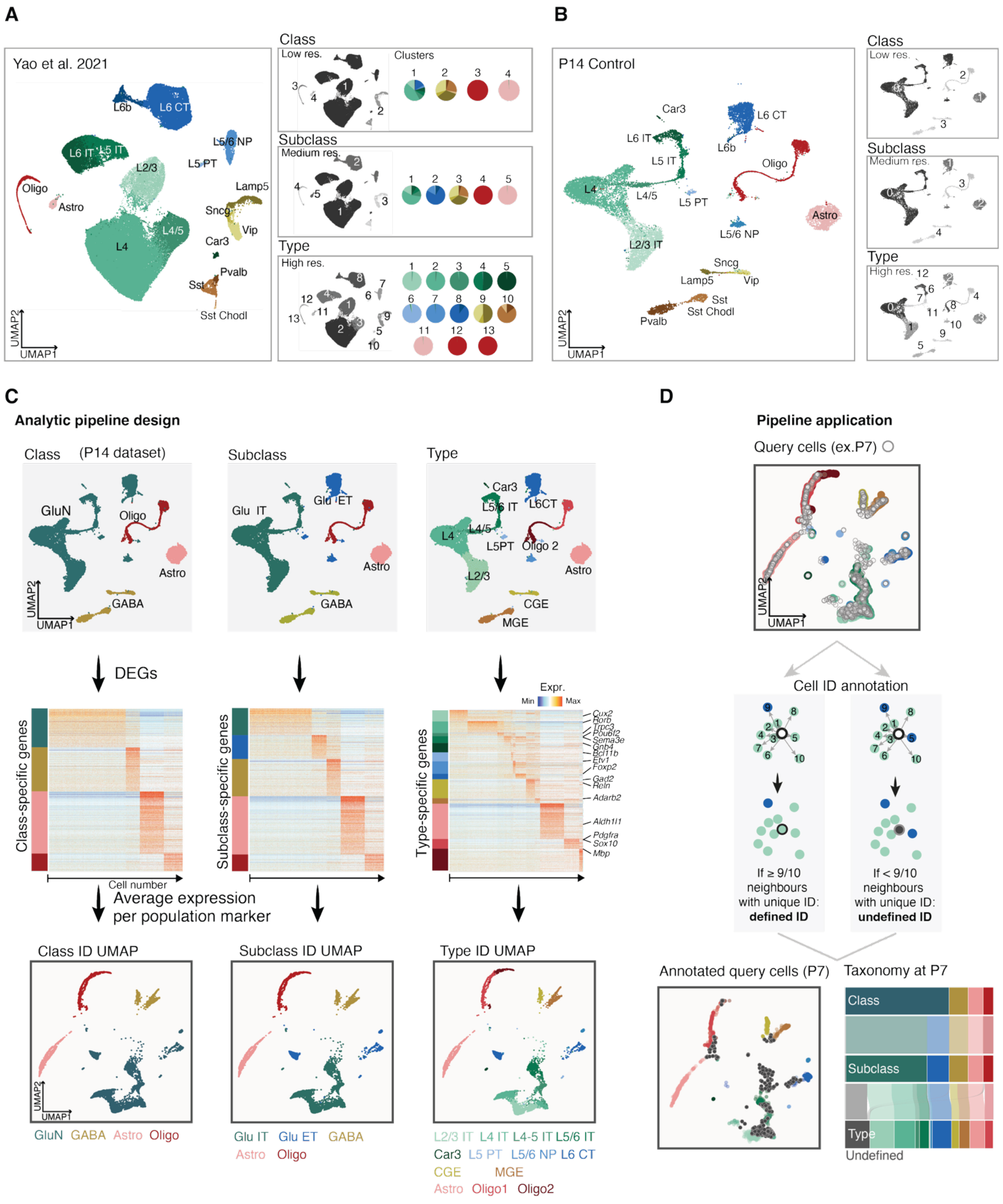

Figure S1: Transcriptomics analysis pipeline for cross-context hierarchical cell classification. A, UMAP representation of the Yao et al, 2021 reference dataset used for the hierarchical tree construction. On the right, the three clustering resolutions used for the classification at in classes, subclasses and types are shown, with the pie charts showing the percentages for each population in the respective clusters. **B,** The same UMAP representations are shown for the reference P14 control used for the identity-based annotation. **C,** representation of the analytical pipeline used to generate the

reference UMAP for each hierarchical level. On the top, the initial UMAPs, followed by the heatmaps showing markers expression for each population (DEGs), and at the bottom the resulting “identity UMAPs” at each hierarchical level. **D,** Schematic description of the analytical pipeline used to annotate cell identities for a query dataset (the example is for P7 in vivo dataset) and the resulting outcomes with Identity UMAP (bottom left) and the hierarchical representation of the population identity annotations with the Sankey plot (bottom right). *Abbreviations*: Glu, glutamatergic neurons; IT, intratelencephalic neurons; ET, extratelencephalic neurons; Astro, astrocytes; Oligo, oligodendrocytes; L6 CT, layer 6 corticothalamic neurons; PT, pyramidal tract neurons; L2/3, layer 2/3; NP, near-projecting neurons; CGE, caudal ganglionic eminence-derived interneurons; MGE, medial ganglionic eminence-derived interneurons; ID, identity; DEGs, differentially expressed genes.

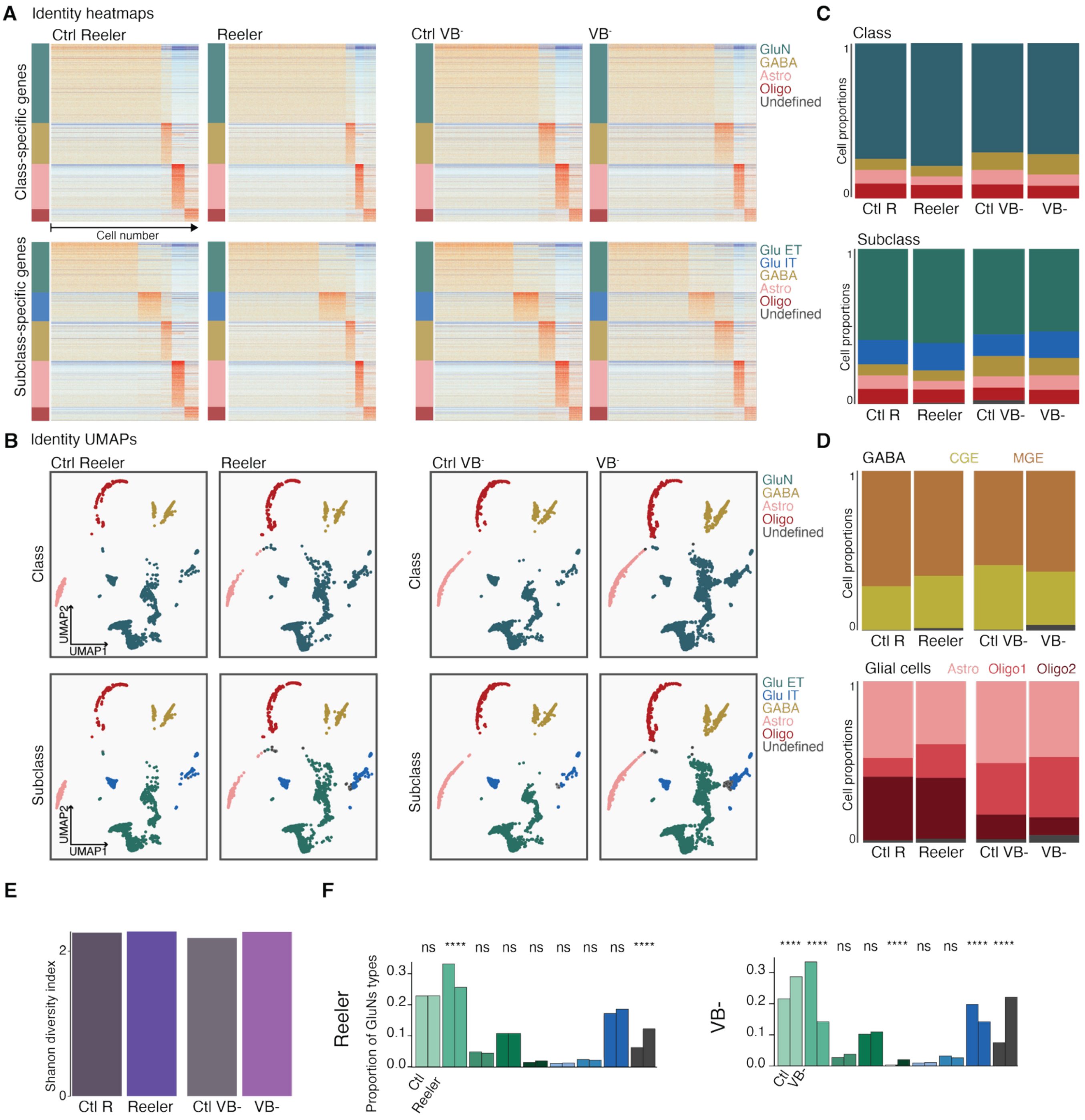

Figure S2: ***In vivo* perturbation of laminar position or thalamocortical input has minimal effect on cellular diversity. A,** Identity heatmaps for cell classes and subclasses across the four conditions. **B,** Identity UMAPs for cell classes and subclasses across the four conditions. **C,** Proportions of cell classes (top) and subclasses (bottom) for the four analyzed conditions. **D,** Proportions of cell types within the GABAergic and glial cell classes for each of the four analyzed conditions. **E,** Shannon index indicating the cell type diversity for all four conditions. **F,** Number of differentially expressed genes per types between mutant and controls. **G,** Proportions of cell types for each genotype. *Abbreviations*: Glu, glutamatergic neurons; IT, intratelencephalic neurons; ET, extratelencephalic neurons; Astro, astrocytes; Oligo, oligodendrocytes; CGE, caudal ganglionic eminence-derived interneurons; MGE, medial ganglionic eminence-derived interneurons.

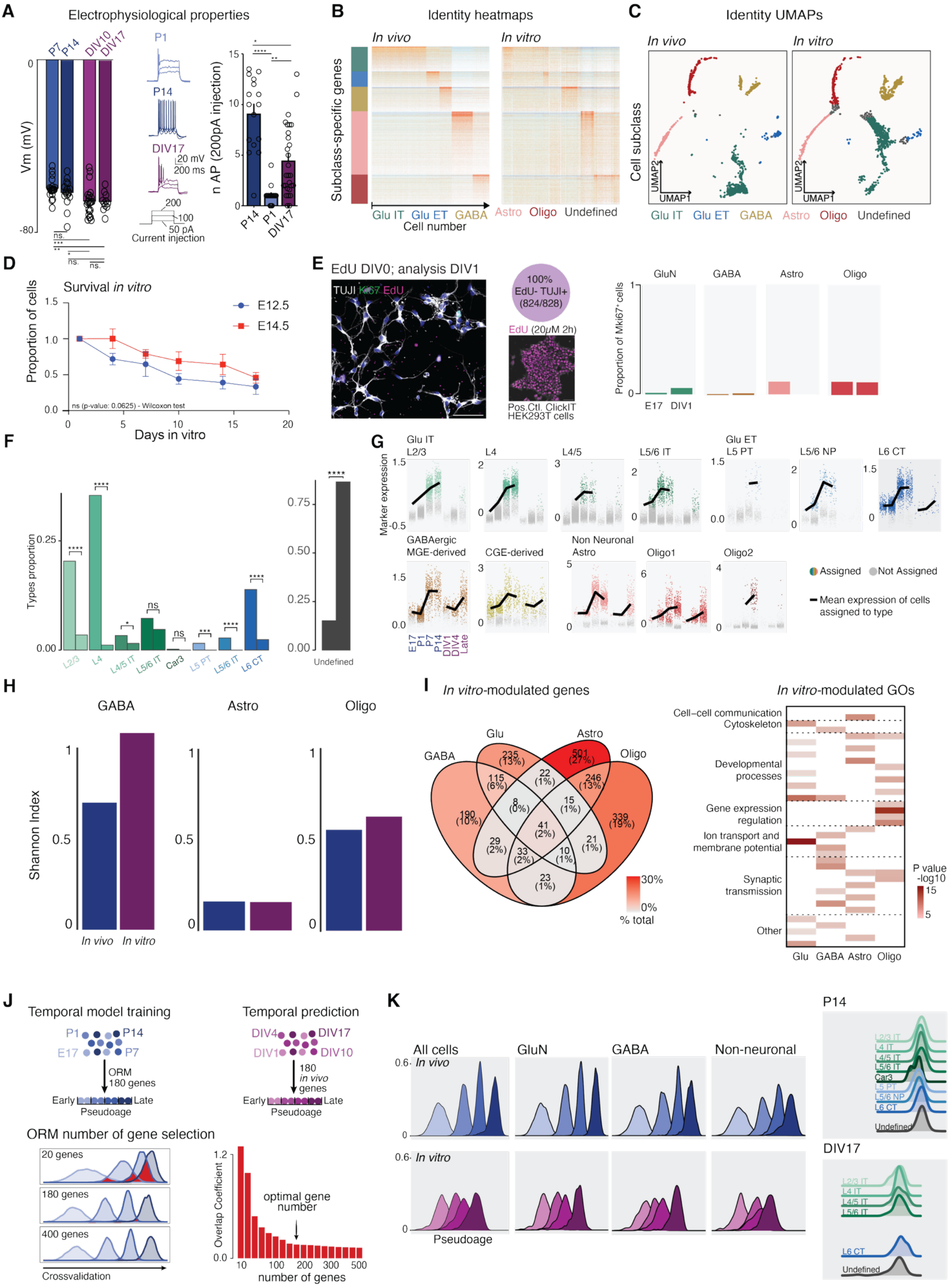

A
Electrophysiological properties
Vm (mV)
P7 P14 DIV10 DIV17
P1
P14
DIV17
20 mV
200 ms
Current injection
n AP (200pA injection)
P14 P1 DIV17
B
Identity heatmaps
In vivo
In vitro
Subclass-specific genes
Cell number
Glu IT Glu ET GABA Astro Oligo Undefined
C
Identity UMAPs
In vivo
In vitro
Cell subclass
UMAP1
UMAP2
Glu IT Glu ET GABA Astro Oligo Undefined
D
Survival in vitro
Proportion of cells
E12.5
E14.5
ns (p-value: 0.0625) - Wilcoxon test
Days in vitro
E
EdU DIV0; analysis DIV1
TUJI Ki67 EdU
100% EdU- TUJI+ (824/828)
EdU (20μM 2h)
Pos.Ctl. ClickIT HEK293T cells
GluN GABA Astro Oligo
Proportion of Mki67+ cells
E17 DIV1
F
Types proportion
L2/3 L4 L4/5 IT L5/6 IT Car3 L5 PT L5/6 IT L6 CT
Undefined
G
Glu IT L2/3
L4
L4/5
L5/6 IT
Glu ET L5 PT
L5/6 NP
L6 CT
Marker expression
GABAergic MGE-derived
CGE-derived
Non Neuronal Astro
Oligo1
Oligo2
E17 P1 P7 P14 DIV1 DIV4 Late
Assigned
Not Assigned
Mean expression of cells assigned to type
H
GABA
Astro
Oligo
Shannon Index
In vivo In vitro
I
In vitro-modulated genes
GABA Glu Astro Oligo
% total
In vitro-modulated GOs
Cell–cell communication
Cytoskeleton
Developmental processes
Gene expression regulation
Ion transport and membrane potential
Synaptic transmission
Other
Glu GABA Astro Oligo
P value -log10
J
Temporal model training
P1 P14 E17 P7
ORM 180 genes
Early Late
Pseudoage
Temporal prediction
DIV4 DIV17 DIV1 DIV10
180 in vivo genes
ORM number of gene selection
20 genes
180 genes
400 genes
Crossvalidation
Overlap Coefficient
optimal gene number
number of genes
K
All cells GluN GABA Non-neuronal
In vivo
In vitro
Pseudoage
P14
DIV17
L2/3 IT L4 IT L4/5 IT L5/6 IT Car3 L5 PT L5/6 NP L6 CT Undefined

Figure S3: Electrophysiological and transcriptomic changes *in vitro*. A, Left, Vm from P7 and P14, DIV10 and DIV17 with example traces; right, number of APs for P14, P1 and DIV17. **B,** Identity heatmaps for subclasses in vivo/in vitro. **C,** Identity UMAPs for subclasses in vivo/in vitro. **D**, Survival curves of E12.5 (blue) and E14.5 (red) electroporated neurons. Error bands: standard error. **E,** immunofluorescence of DIV1 cultures (EdU DIV0) (left) and positive control (middle). Top middle: pie chart with EdU-TUJ1+ proportion. Right, *Mki67*-expressing proportion at E17 *in vivo* and in DIV1 cultures. **F**, Proportion of types for *in vivo/in vitro* conditions. **G**, Average markers expression for each cell type, *in vivo* (left) and *in vitro* (right), across timepoints. Grey dots: Undefined cells. Dark line: mean expression of assigned types. **H,** Shannon index for GABA, astrocyte and oligodendrocyte classes for *in vivo*/*in vitro* conditions. **I,** Left, Venn diagram for DEGs between *in vivo* (P14) and *in vitro* (DIV17) for each class. Right, GO term enrichment heatmap for Biological Process for each class. **J,** Top, schematic of temporal model training using *in vivo* cells and the prediction of *in vitro* cells. Bottom, density plots of prediction values based on number of genes. Left, Overlap between ages (red); Right, sum of overlap. **K,** Left, pseudo-age curves for each timepoint *in vivo* (top) and *in vitro* (bottom) for all cells, or per class. Right, temporal model for P14 and its prediction for DIV17, per cell type. *Abbreviations*: Vm, resting membrane potential; nAP, number of action potentials; Glu, glutamatergic neurons; IT, intratelencephalic neurons; ET, extratelencephalic neurons; Astro, astrocytes; Oligo, oligodendrocytes; CT, corticothalamic neurons; PT, pyramidal tract neurons; L2/3, layer 2/3; NP, near-projecting neurons; CGE, caudal ganglionic eminence; MGE, medial ganglionic eminence; ORM, ordinal regression model; DIV, days in vitro; ns., not significant; GO, gene ontology.

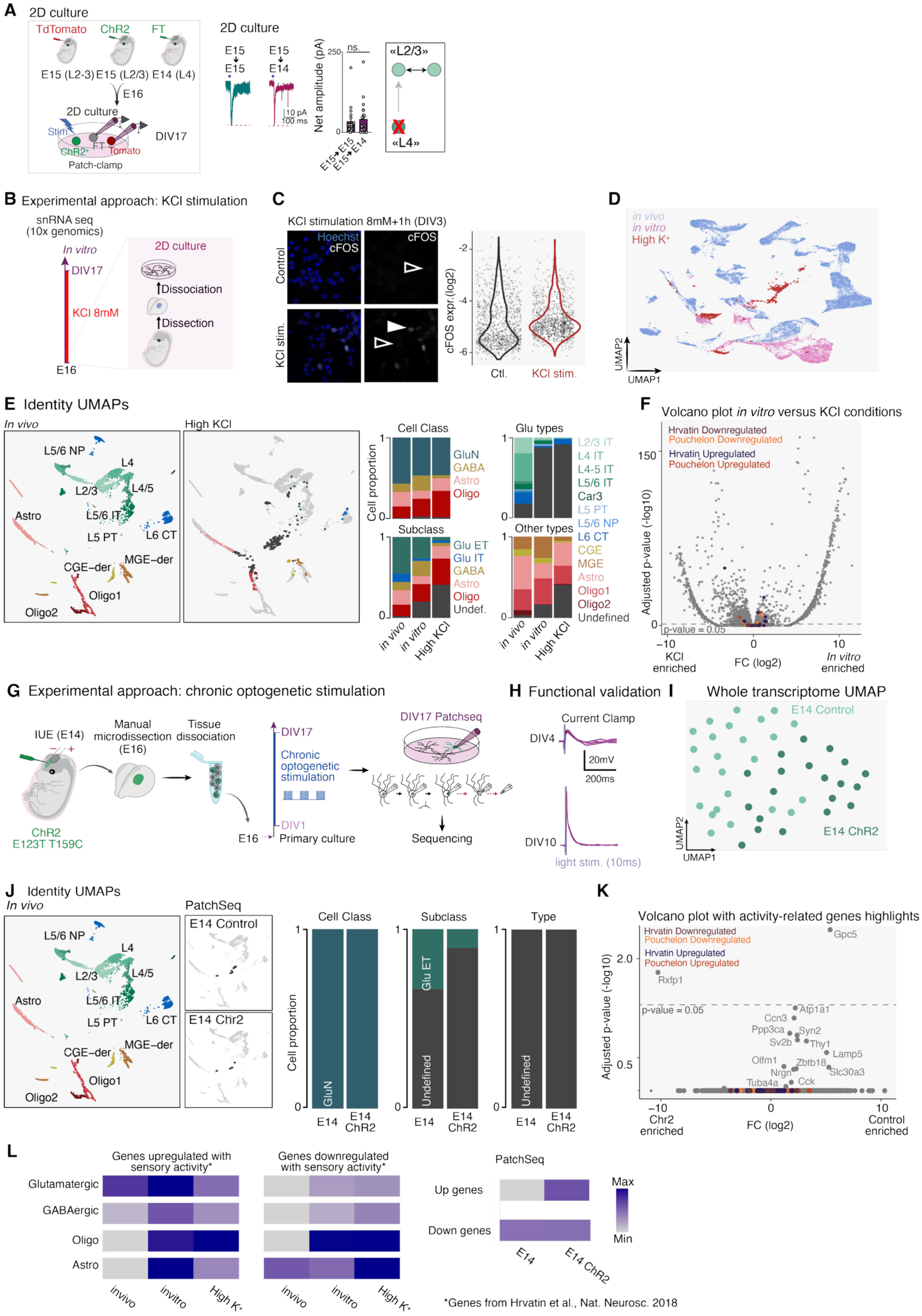
A 2D culture
TdTomato ChR2 FT
E15 (L2-3) E15 (L2/3) E14 (L4)
E16
2D culture
Stim
ChR2+ FT Tomato
DIV17
Patch-clamp
2D culture
E15 → E15
E15 → E14
10 pA
100 ms
Net amplitude (pA)
ns.
«L2/3»
«L4»
B Experimental approach: KCl stimulation
snRNA seq (10x genomics)
In vitro
DIV17
KCl 8mM
E16
2D culture
Dissociation
Dissection
C KCl stimulation 8mM+1h (DIV3)
Hoechst cFOS
cFOS
Control
KCl stim.
cFOS expr.(log2)
Ctl.
KCl stim.
D in vivo
in vitro
High K+
UMAP1
UMAP2
E Identity UMAPs
In vivo
High KCl
L5/6 NP L4 L2/3 L4/5 Astro L5/6 IT L5 PT L6 CT CGE−der MGE−der Oligo1 Oligo2
Cell Class
Cell proportion
GluN GABA Astro Oligo
Subclass
Glu ET Glu IT GABA Astro Oligo Undef.
Glu types
L2/3 IT L4 IT L4-5 IT L5/6 IT Car3 L5 PT L5/6 NP L6 CT
Other types
CGE MGE Astro Oligo1 Oligo2 Undefined
in vivo in vitro High KCl
F Volcano plot in vitro versus KCl conditions
Hrvatin Downregulated
Pouchelon Downregulated
Hrvatin Upregulated
Pouchelon Upregulated
Adjusted p-value (-log10)
p-value = 0.05
KCl enriched
FC (log2)
In vitro enriched
G Experimental approach: chronic optogenetic stimulation
IUE (E14)
Manual microdissection (E16)
Tissue dissociation
ChR2 E123T T159C
DIV17
Chronic optogenetic stimulation
DIV1
E16
Primary culture
DIV17 Patchseq
Sequencing
H Functional validation
Current Clamp
DIV4
20mV
200ms
DIV10
light stim. (10ms)
I Whole transcriptome UMAP
E14 Control
E14 ChR2
UMAP1
UMAP2
J Identity UMAPs
In vivo
PatchSeq
E14 Control
E14 Chr2
Cell Class
Subclass
Type
Cell proportion
GluN
Glu ET
Undefined
E14
E14 ChR2
K Volcano plot with activity-related genes highlights
Hrvatin Downregulated
Pouchelon Downregulated
Hrvatin Upregulated
Pouchelon Upregulated
Gpc5
Rxfp1
p-value = 0.05
Atp1a1
Ccn3
Ppp3ca
Syn2
Sv2b
Thy1
Lamp5
Olfm1
Zbtb18
Nrgn
Slc30a3
Tuba4a
Cck
Adjusted p-value (-log10)
Chr2 enriched
FC (log2)
Control enriched
L
Genes upregulated with sensory activity*
Genes downregulated with sensory activity*
Glutamatergic
GABAergic
Oligo
Astro
invivo invitro High K+
PatchSeq
Up genes
Down genes
E14 E14 ChR2
Max
Min
*Genes from Hrvatin et al., Nat. Neurosc. 2018

Figure S4: **Impact of activity *in vitro***. **A**, Left, experimental scheme. Middle, example of current traces for each condition. Right, bar plot of net amplitudes per condition. **B**, Experimental scheme for chronic KCl stimulation. **C**, Left, immunostaining of control(top) and KCl stimulated cultures(bottom)(empty arrows: $cFOS^-$, full arrow: $cFOS^+$). Violin plot showing intensity of cFOS in control(left) and KCl stimulated cultures(right). **D**, UMAPs of snRNA-seq dataset from Fig. 3C and KCl-stimulated cells, color-coded by condition (top) and age (bottom). **E**, Left, identity UMAPs for P14 condition (from Fig,3) and for DIV17 KCl-stimulated cultures color-coded by type. Right, proportions of cell classes, subclasses and types across *in vivo*, *in vitro* (data from Fig.3) and KCl stimulation. **F**, Volcano plot displaying enriched genes in KCl-stimulated versus control cells. Activity-dependent genes are highlighted. **G**, Experimental scheme for chronic optogenetic stimulation. **H**, Patch-clamp recording at DIV4 and DIV10 of optogenetically-stimulated cells. **I**, Whole transcriptome UMAP of Patch-seq cells color-coded by condition. **J**, Left, identity UMAPs for *in vivo* condition (from Fig. 3), and for Patch-seq *in vitro* cultured and optogenetically-stimulated cells (right). Right, proportions of cell classes, subclasses and types in control versus ChR2-stimulated cells. **K**, Volcano plot displaying enriched genes in ChR2-stimulated cells compared to control Patch-sequenced cells. Activity-dependent genes are highlighted. **L**, Heatmap of sensory activity upregulated or downregulated genes (from Hrvatin et al., 2018) in the different conditions. *Abbreviations*: DIV, day in vitro; ns., not significant; FT, flashtag; snRNA seq, single nucleus RNA sequencing; E, embryonic day; stim., stimulation; expr., expression; Glu, glutamatergic neurons; IT, intratelencephalic neurons; ET, extratelencephalic neurons; Astro, astrocytes; Oligo, oligodendrocytes; L6 CT, layer 6 corticothalamic neurons; PT, pyramidal tract neurons; L2/3, layer 2/3; NP, near-projecting neurons; CGE, caudal ganglionic eminence; MGE, medial ganglionic eminence; FC, fold change; IUE, *in utero* electroporation; Patchseq, patchsequencing; ChR2, channelrhodopsin2.

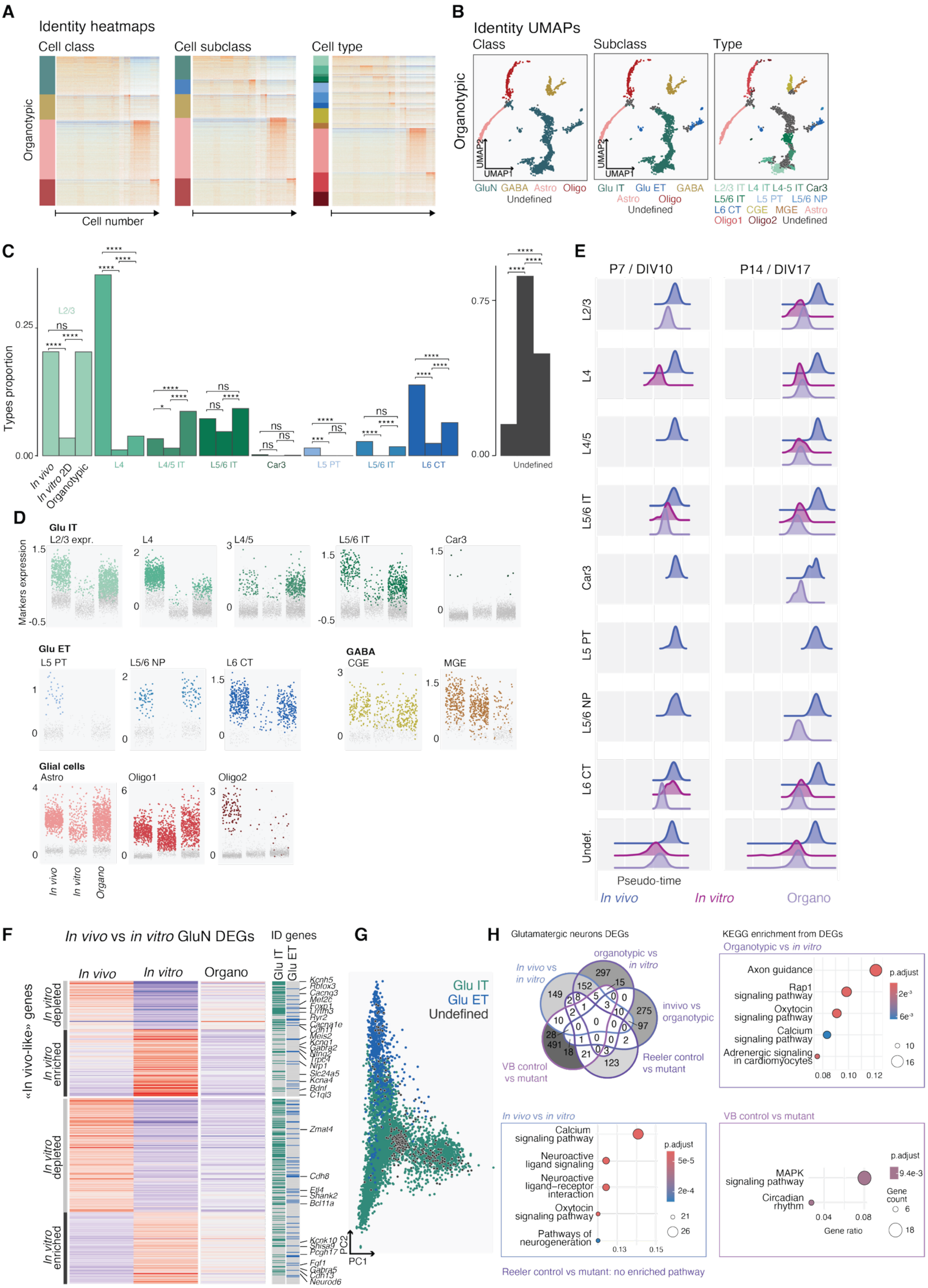
A
Identity heatmaps
Cell class
Cell subclass
Cell type
Organotypic
Cell number
B
Identity UMAPs
Class
Subclass
Type
Organotypic
UMAP1
UMAP2
GluN GABA Astro Oligo
Undefined
Glu IT Glu ET GABA
Astro Oligo
Undefined
L2/3 IT L4 IT L4-5 IT Car3
L5/6 IT L5 PT L5/6 NP
L6 CT CGE MGE Astro
Oligo1 Oligo2 Undefined
C
Types proportion
L2/3
L4
L4/5 IT
L5/6 IT
Car3
L5 PT
L5/6 IT
L6 CT
Undefined
In vivo
In vitro 2D
Organotypic
D
Markers expression
Glu IT
L2/3 expr.
L4
L4/5
L5/6 IT
Car3
Glu ET
L5 PT
L5/6 NP
L6 CT
GABA
CGE
MGE
Glial cells
Astro
Oligo1
Oligo2
In vivo
In vitro
Organo
E
P7 / DIV10
P14 / DIV17
L2/3
L4
L4/5
L5/6 IT
Car3
L5 PT
L5/6 NP
L6 CT
Undef.
Pseudo-time
In vivo
In vitro
Organo
F
In vivo vs in vitro GluN DEGs
ID genes
In vivo
In vitro
Organo
Glu IT
Glu ET
«In vivo-like» genes
In vitro depleted
In vitro enriched
In vitro depleted
In vitro enriched
Kcnh5
Rbfox3
Cacng3
Mef2c
Foxp1
Lrrtm3
Ryr2
Cacna1e
Cdh11
Meis2
Kcnq1
Gabrg2
Ntng2
Trpc4
Nrp1
Slc24a5
Kcna4
Bdnf
C1ql3
Zmat4
Cdh8
Elf4
Shank2
Bcl11a
Kcnk10
Shisa9
Pcgh17
Fgf1
Gabra5
Cdh13
Neurod6
G
Glu IT
Glu ET
Undefined
PC1
PC2
H
Glutamatergic neurons DEGs
In vivo vs in vitro
organotypic vs in vitro
invivo vs organotypic
Reeler control vs mutant
VB control vs mutant
KEGG enrichment from DEGs
Organotypic vs in vitro
Axon guidance
Rap1 signaling pathway
Oxytocin signaling pathway
Calcium signaling pathway
Adrenergic signaling in cardiomyocytes
p.adjust
In vivo vs in vitro
Calcium signaling pathway
Neuroactive ligand signaling
Neuroactive ligand−receptor interaction
Oxytocin signaling pathway
Pathways of neurogeneration
VB control vs mutant
MAPK signaling pathway
Circadian rhythm
Gene count
Gene ratio
Reeler control vs mutant: no enriched pathway

Figure S5: Molecular differences between organotypic and 2D cultures. A, Identity heatmaps across hierarchical levels for organotypic dataset. **B,** Identity UMAPs acrossl hierarchical levels for organotypic dataset. **C**, Proportions of glutamatergic types across In vivo, in vitro 2D and organotypic models. ns., not significant. *: $P < 0.05$. **: $P < 0.01$. ***: $P < 0.001$ ****: $P < 0.0001$. **D**, Average marker expression for each type per cell population for *in vivo* (left), *in vitro* (middle) and organotypic (right). **E**, Temporal model-predicted pseudo-age of organotypic (violet) and in vitro 2D (pink)-harvested cells compared to in vivo (blue) for each type at the indicated timepoints (left, P7 and DIV10; right, P14 and DIV17). **F**, Heatmap showing the *in vivo* vs *in vitro* DEGs average expression (rows), which were expressed (top) and not (bottom) in organotypic cultures-derived glutamatergic neurons. For each condition, the latest timepoint (*in vivo* = P14; *in vitro* = DIV17) is displayed. ID genes of IT and ET neurons are reported (right). The top part of the heatmap is the same of Fig.4E. **G,** PCA analysis of *in vivo* vs *in vitro* DEGs average expression, that were expressed in organotypic culture, per subclass (color-coded). **H**, Venn diagram showing the overlap among the DEGs between organotypic vs in vitro, organotypic vs in vivo, in vivo vs in vitro, Reeler vs control and VB- versus control. White to grey shades indicate increasing number of genes (left top). Five most significantly enriched KEGG pathways in DEGs (left bottom, right).
Abbreviations: Glu, glutamatergic neurons; IT, intratelencephalic neurons; ET, extratelencephalic neurons; Astro, astrocytes; Oligo, oligodendrocytes; L6 CT, layer 6 corticothalamic neurons; PT, pyramidal tract neurons; L2/3, layer 2/3; NP, near-projecting neurons; CGE, caudal ganglionic eminence; MGE, medial ganglionic eminence; DIV, days in vitro; ID, identity.

Table S1
List of genes associated to Class, Subclass and Types. Related to Figure 1.

Table S2
List of Class-defining genes. Related to Figure 1.

Table S3
List of genes used for the temporal model. Related to Figure S3I.

Table S4
Number of nuclei per condition. Related to Methods.

Table S5
List of DEGs between In vivo and In vitro 2D cultures that were restored or not restored in organotypic cultures. Related to Figure 4 and S5.

Table S6
List of DEGs among organotypic vs in vivo, organotypic vs in vitro, in vivo vs in vitro, Control vs Reeler, Control vs VB-. Related to Figure S5H.
